# Antibacterial action against food-borne microorganisms and antioxidant activity of carvacrol-rich oil from *Lippia origanoides* Kunth

**DOI:** 10.1186/s12944-015-0146-7

**Published:** 2015-11-09

**Authors:** Sandra Layse F. Sarrazin, Leomara A. da Silva, Ricardo B. Oliveira, Juliana Divina A. Raposo, Joyce Kelly R. da Silva, Fátima Regina G. Salimena, José Guilherme S. Maia, Rosa Helena V. Mourão

**Affiliations:** Programa de Pós-Graduação em Biodiversidade e Biotecnologia da Amazônia Legal, Universidade Federal do Amazonas, Manaus, 60077-000, AM Brazil; Programa de Pós-Graduação em Recursos Naturais da Amazônia, Universidade Federal do Oeste do Pará, Santarém, 68135-110, PA Brazil; Programa de Pós-Graduação em Química, Universidade Federal do Pará, Belém, 66075-110, PA Brazil; Departamento de Botânica, Instituto de Ciências Biológicas, Universidade Federal de Juiz de Fora, Juiz de Fora, 36036-330, MG Brazil

**Keywords:** Antioxidant and antibacterial activity, Toxicity, Essential oil composition, Carvacrol and thymol

## Abstract

**Background:**

*Lippia origanoides* Kunth from Northeast Brazil is a plant of pleasant odor used by local people as a food seasoning in substitution the oregano where its carvacrol-rich oil has showed significant antimicrobial activity against human pathogens.

**Methods:**

GC and GC-MS analyzed the plant oil composition and its antibacterial activity was evaluated by disk diffusion and microdilution broth methods. The determination of oil antioxidant activity was made by DPPH radical scavenging assay. Oil toxicity was performed on mice.

**Results:**

The main constituents of the oil were carvacrol (47.2 %), thymol (12.8 %), *p*-cymene (9.7 %), and *p*-methoxythymol (7.4 %). The oil was active against the bacteria of *Bacillus cereus*, *B. subtilis*, and *Salmonella typhimurium*, except for *Pseudomonas aeruginosa*. The antioxidant activity has displayed a high dose–response (r^2^ = 0.92), with the inhibition of DPPH radical from 15 to 82 %, at concentrations from 5 to 50 μg/mL, and also by the β-carotene bleaching assay, which showed a high inhibition of 85.2 ± 6.8 %, corresponding to about 80 % of the inhibition of Trolox (93.4 ± 0.7 %), used as a standard. The lethal dose (LD_50_) of the oil was determined in 1673.84 mg mL^−1^.

**Conclusion:**

The results confirmed that the oil of *L. origanoides* could be utilized for the prevention of food bacterial growth, and as an antioxidative agent for retardation of food oxidation process. The oil has low toxicity, allowing its application in the food industry.

**Graphical Abstract:**
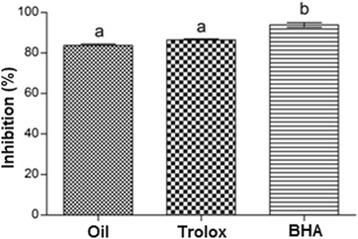
Aerial parts of *Lippia origanoides* Kunth

## Background

Modern techniques of production and conservation of food (high hydrostatic pressure, electromagnetic pulses, active packaging or modified atmosphere systems, natural antimicrobial compounds and biopreservation) have been developed. However, the food security remains an important issue of public health and economics to human society [1]. The growth of pathogenic bacteria and fungi in food can affect its preservation, causing emerging food-borne diseases in different regions of the world. This situation is particularly relevant due to the increased resistance of microorganisms to antimicrobial agents currently used in food preservation. Moreover, the oxidation of lipids in food products can result in the emergence of rancidity and spoilage. Thus, affecting its nutritional quality and giving rise to potentially toxic oxidation products that are not acceptable for the human consumption [2].

The uses of chemical preservatives have been the conventional approach to improving food safety and reducing the oxidation process risks. However, parts of the waste left by these preservatives have shown some degree of toxicity [3]. These findings, along with consumer interest in natural food additives have strengthened the search for natural antioxidants [4]. In this context, the use of essential oils and natural antioxidants has become a promising field of research and potential use after the suspect that synthetic antioxidants such as butyl hydroxyanisole (BHA) and butylated hydroxytoluene (BHT) are potentially harmful to human health [5].

Spices have showed a diversity of volatile constituents with bioactive properties and, among these, many with significant antioxidant activity and use proven against the potential spoilage microorganisms of food [6]. For example, the *Lippia* (Verbenaceae) genus, widely distributed in Central and South America, and Tropical Africa, has presented many species used as food seasoning in different preparations [7]. *Lippia origanoides* Kunth, known in the northern region of Brazil as “salva-do-Marajó”, is a plant of pleasant odor used by local people as a food seasoning in substitution the oregano. Specimens of *L. origanoides* growing wild in areas of the Lower Amazon River, Brazil, have produced carvacrol-rich essential oils with antimicrobial activity against human pathogens of clinical importance [8, 9].

In the present study, the efficacy of the carvacrol-rich oil of *L. origanoides* was evaluated against food-borne bacteria, *Bacillus cereus*, *B. subtilis*, *Pseudomonas aeruginosa*, and *Salmonela typhimurium*, which often are involved in food spoilage process. In addition, the volatiles constituents of the oil were analyzed by GC and GC-MS, and its toxicity and antioxidant capacity were determined.

## Materials and methods

### Solvents and reagents

Solvents (dichloromethane and *n*-hexane) used for the chemical analysis of essential oil were supplied by Merck (Rio de Janeiro, Brazil). Culture media were obtained from Himedia (Mumbai, India). The drugs and reagents (Tween 80 and resazurin, anhydrous sodium sulfate, DPPH, β-carotene and linoleic acid) were obtained from Sigma-Aldrich and Sigma-Vetec (St. Louis, USA and Rio de Janeiro, Brazil). Ampicillin, the standard antimicrobial agent, was supplied by Cefar (São Paulo, Brazil).

### Plant material

Leaves and thin stems (aerial parts) of *L. origanoides* were collected during the growing season, June 2012, in an experimental field of Universidade Federal do Oeste do Pará (UFOPA), Road Everaldo Martins (PA-457), km 26, municipality of Santarém, Pará State, Brazil. The geographical position of the sampled area was determined by GPS, resulting in the coordinates 02°30‘870“S and 54°56’416” W, at an altitude of 52 m above sea level. The collections were performed at morning, 8 to 10 am. Vouchers were deposited in the herbarium of Federal University of Juiz de Fora, Juiz de Fora city, Minas Gerais state, Brazil, under the number CESJ-64029.

### Extraction of the essential oil

Leaves and thin stems were air-dried and submitted to hydrodistillation using a Clevenger-type apparatus (100 g, 3 h). The oil was dried over anhydrous sodium sulfate, and its percentage content was calculated on the basis of the plant dry weight. The moisture contents of the samples were calculated using an Infrared Moisture Balance for water loss measurement. The procedure was performed in triplicate.

### Oil composition analysis

Analysis of the oil was carried on Agilent Technology Equipments: a GC 6890 Plus Series coupled to a selective Mass Spectrometry Detector 5973 and an Auto Sampler 7863, under the following conditions: DB-5 ms (60 m × 0.25 mm; 0.25 mm film thickness) fused-silica capillary column; programmed temperature, 50 °C (5 min in isothermal mode), 150 °C (4 °C/min plus 2 min in isothermal mode), 250 °C (5 °C/min plus 5 min in isothermal mode), 275 °C (10 °C/min plus 15 min in isothermal mode); injector temperature, 250 °C, injection type, split (1 μL) and split flow adjusted to yield 30:1 ratio; carrier gas, helium, with an inlet pressure of 16.5 psi; EIMS, electron energy, 70 eV; temperature of the ion source and transfer line, 230 °C and 285 °C, respectively. The quantitative data regarding the volatile constituents were obtained by peak area normalization using a GC 6890 Plus Series, coupled to FID Detector, operated under similar conditions of the GC-MS system. The retention index was calculated for all the volatiles constituents using a homologous series of n-alkanes (C8–C32, Sigma-Aldrich).

Individual components were identified by comparison of both mass spectrum and GC retention data with authentic compounds that were analyzed and stored in a private library, as well as with the aid of commercial libraries containing retention indices and mass spectra of volatile compounds commonly found in essential oils [10, 11].

### Antimicrobial assays

#### Microorganisms and culture conditions

The strains used in the antimicrobial assays were commercially purchased in the lyophilized form: *Salmonella typhimurium* (CCCD S004), *Pseudomonas aeruginosa* (ATCC 27953), *Bacillus cereus* (CCCD B001) and *Bacillus subutilis* (CCCD B005). The microorganisms were hydrated in nutrient broth (NB) at 37 ± 1 °C, for 24 h. The inocula were prepared by direct inoculation of colonies in 1 ml of sterile saline solution, adjusted to the 0.5 standards of the McFarland scale, corresponding to 1.5 x 10^8^ UFC/ml [12].

### Agar disk diffusion method

The antimicrobial activity of the oil was performed by a standard method of agar disc diffusion [12]. Mueller Hinton Agar (MHA) was used as a growth medium for microorganisms. Filter paper disks of 6 mm diameter, containing 10 μl of the undiluted oil, were pressed lightly against the surface of the agar, which was previously seeded with the tested microorganisms. After 30 min at room temperature, the dishes were incubated at 37 ± 1 °C, for 24 h. At the end of the test period, the diameter of the inhibition zone formed over the agar was measured in millimeters. Ampicillin (10 μg) was used as the positive standard. The test was performed in triplicate, and the values obtained were compared to the positive control.

### Broth microdilution method

The determination of minimum inhibitory concentration (MIC) and minimum bactericidal concentration (MBC) [13]. The initial test concentration was prepared by dissolving 10 μl of oil in 1 ml of Tween 80 (1.0 %). From this stock solution, concentrations from 0.07 to 5.0 μl/ml were equally prepared by serial dilution (dilution factor 1:1) using Mueller-Hinton Broth (MHB) as the solvent. The inoculum was standardized according to the scale of 0.5 MacFarland (dilution factor 1:10, sterile saline solution) to obtain the final concentration of 1.5 × 10^4^ CFU/ml. The tests were performed in 96 well plates, where each well received 90 μl of the specific concentration of oil, 90 μl of sterile MHB and 20 μl of the inoculum. Ampicillin (positive control) was diluted with sterile distilled water to obtain concentrations ranging from 0.1 to 46.9 μg/ml. The microbial growth control, the control of sterility of the medium and the control of solvent were done simultaneously. Each well had a final volume of 200 μl. The inhibition of growth of bacteria was revealed by the addition of resazurin sterile solution (20 μl, 0.02 %, w/v) and re-incubation for 3 h. MIC, which is defined as the lowest concentration of oil capable of inhibiting the growth of microorganisms, was determined by the permanence of blue coloration in the wells. A change of color from blue to red (due to the reduction of dye) indicated the bacterial growth. The wells that showed no apparent growth were selected to evaluate the MBC, which was determined by the absence of microbial growth on plates containing MHA. The tests were performed in triplicate.

### Antioxidant assay

The antioxidant activity of the *L. origanoides* oil was evaluated by DPPH radical scavenging assay and β-carotene bleaching assay, using methodologies previously described, with some modifications [14].

#### DPPH radical scavenging assay

A stock solution of DPPH reagent (0.5 mM) was prepared in methanol. The solution was diluted with methanol (60 μM approx.) to yield an initial absorbance of 0.62 ± 0.02 at 517 nm. The reaction mixture was composed of 1950 μl of DPPH solution and 50 μl of the oil, diluted with different proportions of methanol. For each sample, a methanol blank was also used. Trolox (6-hydroxy-2,5,7,8-tetramethylchroman-2-carboxylic acid) was used as standard antioxidant. The absorbance was measured after 10 and 60 min for the samples and Trolox, respectively. The radical-scavenging activity was calculated by the DPPH-inhibition percentage according to the equation: I% = 100 (A-B)/A, where A and B are the blank and sample absorbance values, at the end of the reaction. Linear regression determined the concentration of antioxidant required for 50 % scavenging of DPPH radicals (half-maximal effective concentration or EC_50_). The analysis was performed in triplicate and the result presented as mean value ± standard deviation.

#### β-carotene bleaching assay

A stock solution of *β*-carotene and linoleic acid mixture was prepared as following: 0.2 mg of β*-*carotene was dissolved in 1 ml of chloroform (HPLC grade) and, then, 20 μl of linoleic acid and 265 μl of Tween 40 were added. Chloroform was completely removed using a vacuum evaporator. Then, 100 ml of ultra-pure water, saturated with oxygen, was added with vigorous shaking. An aliquot of 2500 μl of this reaction mixture was dispensed into test tubes. Portions of 200 μl of the samples, prepared at 1.0 mg/l of ethanol were added, and the emulsion system was incubated at 50 °C. The same procedure was repeated with BHA, Trolox and the control (ethanol). The absorbance of these solutions was recorded and monitored at intervals of 15 min during 120 min, at 470 nm. The antioxidant activity (AA%) was calculated in terms of percent inhibition relative to the control using the Equation: AA% = [A_0_-A_120_]/[B_0_- B_120_] x 100, where A_0_, A_120_, B_0_, and B_120_ are the absorbance of the sample and the control at the beginning and end of the reaction.

### Acute toxicity (LD_50_)

Male Swiss mice were used, aged 60 ± 2 days, body mass index between 35 and 47 g. Mice were divided into six groups of twelve. Each group fasted for 12 h received oral doses of the oil between 100 and 3000 mg kg^−1^ according to the body weight of each animal. Initially, the animals were placed in cages and observed for 4 h to analyze the following characteristics: aggressiveness, motor activity and lethargy, lack of appetite, runny nose, piloerection, urination, diarrhea, and convulsions. Then, the animals were observed for an additional period of 24, 48 and 72 h, and remained under the same conditions (food and water *ad libitum*, controlled temperature (24 ± 2 °C), 12 h light/dark cycle) for 14 days to check possible occurrence of death. A separate group of animals served as a control group. The LD_50_ was calculated by semi-logarithmic interpolation, placing the values corresponding to the probabilistic percentage of deaths in ordinate axis and the doses administered, in the abscissa axis [15].

### Statistical analyses

The data were subjected to analysis of variance (ANOVA) and the differences between means were determined by Tukey test (p ≤ 0.05), using GraphPad Prism 5.0 and StatPlus 2009.

## Results and discussion

### Oil composition

The yield content of the oil of *L. origanoides* was 1.3 %, and it was analyzed by GC and GC-MS. Thirty-three volatile constituents were identified, and they are listed in Table 1. Carvacrol (47.2 %), thymol (12.8 %), *p*-cymene (9.7 %) and *p*-methoxythymol (7.4 %) were the main components.

**Table 1:** Oil composition (%) of *Lippia origanoides*

N°	Constituents	RI_Calc._	RI_Lit._	Oil %
01	(*Z)*-Hexen-3-ol	854	859	0.2
02	α-Thujene	926	924	0.6
03	α-Pinene	934	932	0.4
04	1-Octen-3-ol	976	974	0.1
05	Myrcene	990	988	1.1
06	α-Terpinene	1016	1014	0.5
07	***p*** **-Cymene**	1025	1020	**9.7**
08	Limonene	1026	1024	0.2
09	1,8-Cineole	1032	1026	1.3
10	γ-Terpinene	1056	1054	0.2
11	Linalool	1098	1095	2.9
12	Umbellulone	1169	1167	0.3
13	Terpinen-4-ol	1176	1174	1.5
14	Thymol methyl ether	1234	1232	1.3
15	Thymol/carvacrol isomer (MW = 150)	1282		0.4
16	**Thymol**	1292	1289	**12.8**
17	**Carvacrol**	1299	1298	**47.2**
18	Thymol acetate	1351	1349	0.4
19	Carvacrol acetate	1372	1370	0.6
20	Geranyl acetate	1382	1379	0.6
21	(*E*)-Caryophyllene	1418	1416	2.3
22	*trans*-α-Bergamotene	1434	1432	0.2
23	α-Humulene	1455	1452	0.3
24	***p*** **-Methoxythymol**	1487	1484	**7.4**
25	β-Bisabolene	1506	1505	0.3
26	(*Z*)-α-Bisabolene	1508	1506	0.2
27	*p*-Methoxycarvacrol (tent.)	1555		1.3
28	Caryophyllene oxide	1584	1582	1.6
29	2-phenylethyl tyglate	1587	1584	0.2
30	Humulene epoxide II	1611	1608	0.2
31	α-Eudesmol	1656	1653	0.1
32	α-Bisabolol	1686	1685	0.2
33	Unidentified sesquiterpenes			1.2
Total				**97.8**

*RI*_*Calc*._ Retention index calculated on DB-5 ms capillary column, using a homologous; series of *n*-alkanes (C_8_-C_32_). RI_Lit._ = Retention index from literature [10]; Bold data: The main constituents of the oil.

The seasonal variation of the essential oil of a specimen of *L. origanoides* that occurs in lower Amazon River, the same sampling area of this study, was recently reported [9]. The main constituents found were carvacrol (rainy season: 43.5 %, dry season: 41.4 %), thymol (rainy season: 10.7 %, dry season: 10.6 %), *p*-cymene (rainy season: 9.8 %, dry season: 10.0 %) and *p*-methoxythymol (rainy season: 9.6 %, dry season: 10.4 %. Therefore, with a very similar oil composition.

Some chemotypes for the oil of *L. origanoides* have been described. The leaf oils of three specimens occurring in the localities of Cabeceiras, Campo Maior and José de Freitas, Piauí state, Brazil, showed similar composition with carvacrol as the major component (*ca*. 43 %) [16]. *Lippia schomburgkiana* Schauer, a synonymous species of *L. origanoides* Kunth, collected in Anapurus, Maranhão state, Brazil, yielded an oil dominated by 1,8-cineole (64.1 %) [17]. The oil obtained from a specimen sampled in Oriximiná, Pará state, Brazil, also presented carvacrol (*ca*. 38 %) as the main constituent [8]. Oils from three specimens that grow in the Departments of Santander, Cauca, Nariño and Boyacá, Colombia, have showed different composition: (1) oil with predominance of thymol (*ca*. 56 %), (2) an oil dominated by carvacrol (*ca*. 40 %), and (3) oil where *p*-cymene (12 %) was the principal constituent [18]. Finally, an oil rich in (E)-methyl cinnamate (52.4 %) and (E)-nerolidol (23.2 %) was reported for a specimen of *L. origanoides* with occurrence in the National Forest of Carajás, Parauapebas, Pará state, Brazil [19].

The genus *Lippia*, with more than fifty reported essential oils, is well known for its aromatic character [20]. *Lippia* species that occur in Brazil have shown a wide variation in the oil composition with the description of several chemotypes. *Lippia alba* (Mill.) N.E. Br collected in Pará and Ceará states, with the types citral, carvone and 1,8-cineole [21]; and *Lippia glandulosa* Schauer that grows wild in Roraima state, with the types thymol and β-caryophyllene [22]. Large amounts of thymol and carvacrol have been also identified in *Lippia gracilis* Schauer and *Lippia sidoides* Cham. from Northeast Brazil [23, 24].

Differences in essential oils composition can be attributed to the influence of phenological stage and the environmental factors for the plant collection sites. These phenomena may significantly alter the biochemical pathways and physiological processes that regulate the metabolism of the plant and, therefore, the biosynthesis of essential oils [25].

### Antimicrobial activity

Some species of *Lippia* are known for essential oil production that exhibit antimicrobial potential. Among these, *L. chevalieri* Moldenke and *L. multiflora* Moldenke [26], *L. javanica* Spreng. [27], *L. sidoides* Cham. [23], *L. alba* (Mill.) N. E. Brown [28], *L. gracilis* Schauer [24], *L. palmieri* S. Wats [29] and *L. origanoides* Kunth [9]. In the present study, the antimicrobial activity of a carvacrol-rich oil of *L. origanoides* was evaluated against some food-borne bacteria, by using the methods of disk diffusion and microdilution broth. The diameter of microorganism growth inhibition zone, the minimum inhibitory concentration (MIC) and minimum bactericidal concentration (MBC) are summarized in Table 2.

**Table 2:** Results of antibacterial activity of the oil of *L. origanoides* against food-borne bacteria

Bacteria	Oil			Ampicillin		
	DD (mm)	MIC	MBC	DD	MIC	MBC
	10 μl	μL/ml	μL/ml	10 μg	μg/ml	μg/ml
*Salmonella typhimurium*	27.9 ± 0.4*	1.25	10	19.1 ± 0.2	1.5	2.9
CCCD S004						
*Bacillus cereus*	50.9 ± 0.6*	0.62	>20	23.4 ± 0.2	1.5	5.8
CCCD B001						
*Bacillus subtilis*	35.8 ± 0.3*	1.25	1.25	21.0 ± 0.2	0.18	0.18
CCCD B005						
*Pseudomonas aeruginosa*	na	nt	nt	na	nt	nt
ATCC 27953						

*p ≤ 0,05 - Significance relative to the control; *DD* disk diffusion; *MIC* Minimum Inhibitory Concentration; *MBC* Minimum Bactericidal Concentration; *na* not active; *nt* not tested

The oil of *L. origanoides* exhibited significant antimicrobial activity (p ≤ 0.05) when compared to positive control. The inhibition zone ranged from 27.9 to 50.9 mm, showing strong antimicrobial activity against the tested microorganisms, except for the bacteria *Pseudomonas aeruginosa*, that it proved to be resistant to the oil. The largest zone of inhibition was observed for *Bacillus cereus* CCCD B001 (50.9 mm, 0.62 μl/ml), followed by *Bacillus subtilis* CCCD B005 (35.8 mm, 1.25 μl/ml) and *Salmonella typhimurium* CCCD S004 (27.9 mm, 1.25 μl/ml). Also in this order, the strongest antibacterial activity of microorganisms tested. In general, the antimicrobial activity of essential oils is classified into different levels: weak activity (inhibition zone ≤ 12 mm), moderate activity (12 mm < inhibition zone < 20 mm) and strong activity (inhibition zone ≥ 20 mm) [30].

It is important to consider that the percentage of carvacrol and thymol in the oil of *L. origanoides* is 60 %. The antimicrobial activity of carvacrol and thymol has been reported [31, 32]. Carvacrol and thymol are monoterpene phenols biosynthesized from γ-terpinene through *p*-cymene, and these latter compounds are always present in the same oil. The only difference in the formula of thymol and carvacrol is the position of the hydroxyl group on the phenyl ring [31]. However, independently of the position of the hydroxyl group, carvacrol and thymol have showed similar antimicrobial potential [31]. The mechanism of action of carvacrol and thymol involves the disruption of the cell membrane and subsequent escape of cytoplasmic contents [33]. In Gram-negative bacteria, carvacrol and thymol provoke the disintegration of the external membrane, liberating lipopolysaccharides and increasing the permeability of the cytoplasmic membrane [2].

In the analysis of antimicrobial agents, the plants can have advantages compared to synthetic drugs because many of its constituents can have similar biological effects, acting synergistically and increasing its therapeutic effect. The antimicrobial activity of essential oils may depend on only one or two of the major constituents that make up the oil. Synergism (partial or total) between carvacrol and thymol has been reported to control the growth of microorganisms [34]. However, evidence indicate that the inherent activity of essential oils may not depend exclusively on the ratio in which the main active constituents are present, but also from the interactions between these and the minor constituents in the oils [1]. For example, *p*-cymene (biosynthetic precursor of carvacrol and thymol), which occurs in the oil of *L. origanoides* with 9.7 %, is not an efficient antimicrobial agent when used alone, but together carvacrol and thymol it can potentiate the action of the oil to promoting the cytoplasmic membrane expansion and facilitating the antimicrobial action of these monoterpene phenols [31]. In addition, linalool (monoterpene alcohol, 2.9 % in the oil) can also contribute synergistically to the antibacterial activity of the oil of *L. origanoides* since it has previously proven its antimicrobial action [35].

Thus, the above results have highlighted the effectiveness of the oil of *L. origanoides* against microorganisms that are often involved in the deterioration and/or contamination of food.

### Antioxidant activity

In present work, the antioxidant activity of the oil of *L. origanoides* was evaluated by two different assays: DPPH radical scavenging and β*-*carotene bleaching. The oil interacted with the DPPH by transfer of electron or hydrogen, neutralizing its free radical character [14]. The oil was able to promote the scavenging of DPPH radical, displaying a high dose–response (r2 = 0.92), with inhibition varying from 15 to 82 %, at concentrations from 5 to 50 μg/ml. The half-maximal effective concentration (EC_50_) was 23.0 ± 1.5 μg/ml, calculated by linear regression (p < 0.05), representing a significant antioxidant activity and comparable to the standard Trolox (3.0 ± 0.3 μg/ml), as can be seeing in Table 3. EC_50_ values lower than 30 μg/ml indicates a significant potential for radical scavenging [36].

**Table 3:** Antioxidant activity of essential oil of *L. origanoides* in DPPD assay

Samples	Concentration	Inhibition	EC_50_
	(μg/ml)	(%)	(μg/ml)
	50.0	81.5 ± 4.9	
	40.0	73.6 ± 3.5	
Oil	30.0	68.0 ± 4.9	23.0 ± 1.5
	20.0	54.5 ± 2.6	
	10.0	31.6 ± 0.9	
	5.0	15.1 ± 3.5	
	5.0	80.3 ± 5.4	
	4.0	64.2 ± 4.6	
Trolox	3.0	53.8 ± 4.6	3.0 ± 0.3
	2.0	36.4 ± 5.2	
	1.0	17.2 ± 2.2	

**Fig. 1:**
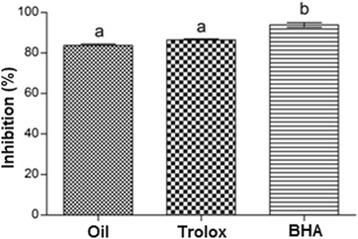
Antioxidant activity for the oil of *Lippia origanoides* by β-carotene/linoleic acid assay. ^a,b^ Values with same letter are not statiscally different at the < 0.05 level (Tukey’s test)

The β-carotene bleaching assay has evaluated by inhibiting the activity of free radicals generated during linoleic acid peroxidation, in the presence of the oil. The rate of β-carotene bleaching was slowed down in the presence of the oil. Thus, it was possible to evaluate the antioxidant activity of the oil of *L. origanoides* in comparison with the synthetic antioxidants BHA and Trolox. The reaction was monitored for 120 min and inhibition of oil oxidation was 85.2 ± 6.8 %. This value was about 80 % of the inhibition value observed for Trolox (93.4 ± 0.7 %). The ability of the oil of *L. origanoides* to protect the discoloration of *β*-carotene about Trolox and BHA is displayed in Fig. 1. These results are comparable to those obtained for the oils of *Satureja spicigera* (C. Koch) Boiss. (carvacrol 42.5 %) and *S. cuneifolia* Ten. (carvacrol 67.1 %), that had promoted the inhibition of 81.7 and 93.7 %, respectively when used the β-carotene bleaching assay [37].

The antioxidant activity of a carvacrol-rich oil of *L. origanoides*, collected in the Department of Santander, Colombia, using the ABTS radical cation, was previously reported [38], and this oil showed less activity in comparison with synthetic antioxidants as BHA and α-tocopherol, as well high activity when faced to BHT. Also, another carvacrol-rich oil of *L. origanoides* cultivated in Feira de Santana, Bahia state, Brazil, showed that plants in growth stage have displayed better antioxidant activity than grown plants [39].

The antioxidant action is a complex process that is usually occurring by various mechanisms. Thus, the result of a single assay for essential oil can give only a relative suggestion of its antioxidant properties in food matrices and must be interpreted with some caution. Moreover, the essential oil is a complex chemical mixture, composed of tens of components with different functional groups, polarity, and chemical behavior. Thus, depending on the used test, this can lead to diverse results and the difficulty to explain the activity described above [40]. For this reason, the DPPH radical and β-carotene bleaching assays were used in analyzing the antioxidant activity of the oil of *L. origanoides* and, as seen above, the results of both tests were effective and complementary.

### Acute toxicity

The determination of lethal dose has indicated low toxicity (LD_50_, 1673.84 mg kg^−1^) to the oil of *L. origanoides*. In the first 4 h of observation, only those animals that received a dose of 3000 mg kg^−1^ exhibited lethargy and piloerection. In the other doses tested, no change was observed in the physiological or behavioral parameters. In a previous study, physiological or behavioral changes in mice were not observed, after oral administration of the oil at doses of 30, 60 and 120 mg kg^−1^ [41].

## Conclusion

The essential oil of *L. origanoides* was efficient in the control of the growth of microorganisms as a natural antioxidant product. The results confirm the alternative use of the oil in the prevention of foodborne bacteria, as well as an agent to promote delay in the oxidation process. Therefore, the oil could have a viable application as a food preservative, assisting in the control of contamination by microorganisms and the oxidation process of processed foods.
